# Epilepsy Personal Assistant Device—A Mobile Platform for Brain State, Dense Behavioral and Physiology Tracking and Controlling Adaptive Stimulation

**DOI:** 10.3389/fneur.2021.704170

**Published:** 2021-07-29

**Authors:** Tal Pal Attia, Daniel Crepeau, Vaclav Kremen, Mona Nasseri, Hari Guragain, Steven W. Steele, Vladimir Sladky, Petr Nejedly, Filip Mivalt, Jeffrey A. Herron, Matt Stead, Timothy Denison, Gregory A. Worrell, Benjamin H. Brinkmann

**Affiliations:** ^1^Bioelectronics Neurophysiology and Engineering Laboratory, Department of Neurology, Mayo Clinic, Rochester, MN, United States; ^2^Cognitive Systems and Neurosciences, Czech Institute of Informatics, Robotics and Cybernetics, Czech Technical University in Prague, Prague, Czechia; ^3^Department of Physiology and Biomedical Engineering, Mayo Clinic, Rochester, MN, United States; ^4^School of Engineering, University of North Florida, Jacksonville, FL, United States; ^5^Division of Engineering, Mayo Clinic, Rochester, MN, United States; ^6^Faculty of Biomedical Engineering, Czech Technical University in Prague, Kladno, Czechia; ^7^Department of Neurological Surgery, University of Washington, Seattle, WA, United States; ^8^Engineering Sciences and Clinical Neurosciences, Oxford University, Oxford, United Kingdom

**Keywords:** epilepsy, deep brain stimulation, implantable devices, neuromodulation, seizure detection, seizure prediction, wearables

## Abstract

Epilepsy is one of the most common neurological disorders, and it affects almost 1% of the population worldwide. Many people living with epilepsy continue to have seizures despite anti-epileptic medication therapy, surgical treatments, and neuromodulation therapy. The unpredictability of seizures is one of the most disabling aspects of epilepsy. Furthermore, epilepsy is associated with sleep, cognitive, and psychiatric comorbidities, which significantly impact the quality of life. Seizure predictions could potentially be used to adjust neuromodulation therapy to prevent the onset of a seizure and empower patients to avoid sensitive activities during high-risk periods. Long-term objective data is needed to provide a clearer view of brain electrical activity and an objective measure of the efficacy of therapeutic measures for optimal epilepsy care. While neuromodulation devices offer the potential for acquiring long-term data, available devices provide very little information regarding brain activity and therapy effectiveness. Also, seizure diaries kept by patients or caregivers are subjective and have been shown to be unreliable, in particular for patients with memory-impairing seizures. This paper describes the design, architecture, and development of the Mayo Epilepsy Personal Assistant Device (EPAD). The EPAD has bi-directional connectivity to the implanted investigational Medtronic Summit RC+S^TM^ device to implement intracranial EEG and physiological monitoring, processing, and control of the overall system and wearable devices streaming physiological time-series signals. In order to mitigate risk and comply with regulatory requirements, we developed a Quality Management System (QMS) to define the development process of the EPAD system, including Risk Analysis, Verification, Validation, and protocol mitigations. Extensive verification and validation testing were performed on thirteen canines and benchtop systems. The system is now under a first-in-human trial as part of the US FDA Investigational Device Exemption given in 2018 to study modulated responsive and predictive stimulation using the Mayo EPAD system and investigational Medtronic Summit RC+S^TM^ in ten patients with non-resectable dominant or bilateral mesial temporal lobe epilepsy. The EPAD system coupled with an implanted device capable of EEG telemetry represents a next-generation solution to optimizing neuromodulation therapy.

## Introduction

Drug-resistant epilepsy is one of the most common neurological disorders, affecting almost 1% of the population worldwide (1, 2). Many people living with epilepsy continue to have seizures despite anti-epileptic medication therapy (3, 4), and for them, resective surgery and neuromodulation therapy are the primary therapeutic options. Resective surgery can be attempted if a focal seizure onset zone can be identified, typically via invasive EEG monitoring, and if this area can be removed without causing a functional deficit. Although often effective, brain resection is irreversible, and for many patients, seizures eventually reoccur.

Neuromodulation therapy for epilepsy has grown in prevalence following FDA approvals for responsive neurostimulation (RNS) (5) in 2013 and deep brain stimulation (DBS) (6) in 2018. While these approaches effectively reduce seizures, long-term seizure freedom is rare with these methods. Additionally, optimization of therapeutic parameters, including stimulation amplitude, rate, and pulse width, is a very slow process, and optimal therapeutic effect is only achieved after many years (6, 7). Due to the poor reliability of self-reported seizure diaries (8), physicians may have difficulty knowing how effective a given set of stimulation settings is at suppressing or preventing seizures. Current devices have very limited capability to record and report seizure activity measures (typically EEG activity). However, limited objective measures of seizure rates and epileptiform activity are currently available and have produced profound insights already (9, 10).

Seizure predictions could potentially be used to adjust neuromodulation therapy to prevent the onset of a seizure and prompt medication therapy or to empower patients to avoid sensitive activities during high-risk periods (11, 12). An experimental device (NeuroVista SAS) was preclinically trialed provided clear proof of concept and validation of the value of long-term objective EEG data and seizure forecasts (13–15). However, the device did not progress to approval for clinical use and is no longer available, leaving an unmet need for a next-generation device with long-term EEG and seizure forecasting capabilities. Medtronic Inc. recently designed a novel experimental device with EEG telemetry and therapy modulation capabilities (16). The investigational Medtronic Summit RC+S^TM^ system was developed to telemeter EEG, provide on-device seizure detections, and modulate stimulation therapy based on either on-board EEG analytics or analytics on an associated mobile computer. A full-featured analytics platform is needed to configure sensing and analytics on the device, manage device connectivity and data telemetry, provide distributed analytics for modulation of stimulation, and interact with subjects to deploy such capabilities successfully.

All software and components must be developed in compliance with international engineering standards (in particular ISO 60601) and a design control process compliant with United States federal regulations (specifically Title 21, section 820.20) to use such a system in human subjects. Significant preclinical testing and comprehensive verification and validation testing regimen must be employed to ensure system safety and quality.

As devices become increasingly interconnected and operate in the context of analytical and cloud computational systems, compliance with regulations governing software as a medical device is required, and developing regulations around machine learning (17, 18) must be included to augment the traditional regulatory framework around medical device development. At the core of the design process, user needs, and requirements are translated into system design and implemented with clear documentation and a rigorous process for testing, defect correction, and design updates. The complexity and required skill set for this is often missing in research lab environments, and likely contributes to the many barriers to translation of benchtop discoveries to clinical practice (19).

**Figure 1:**
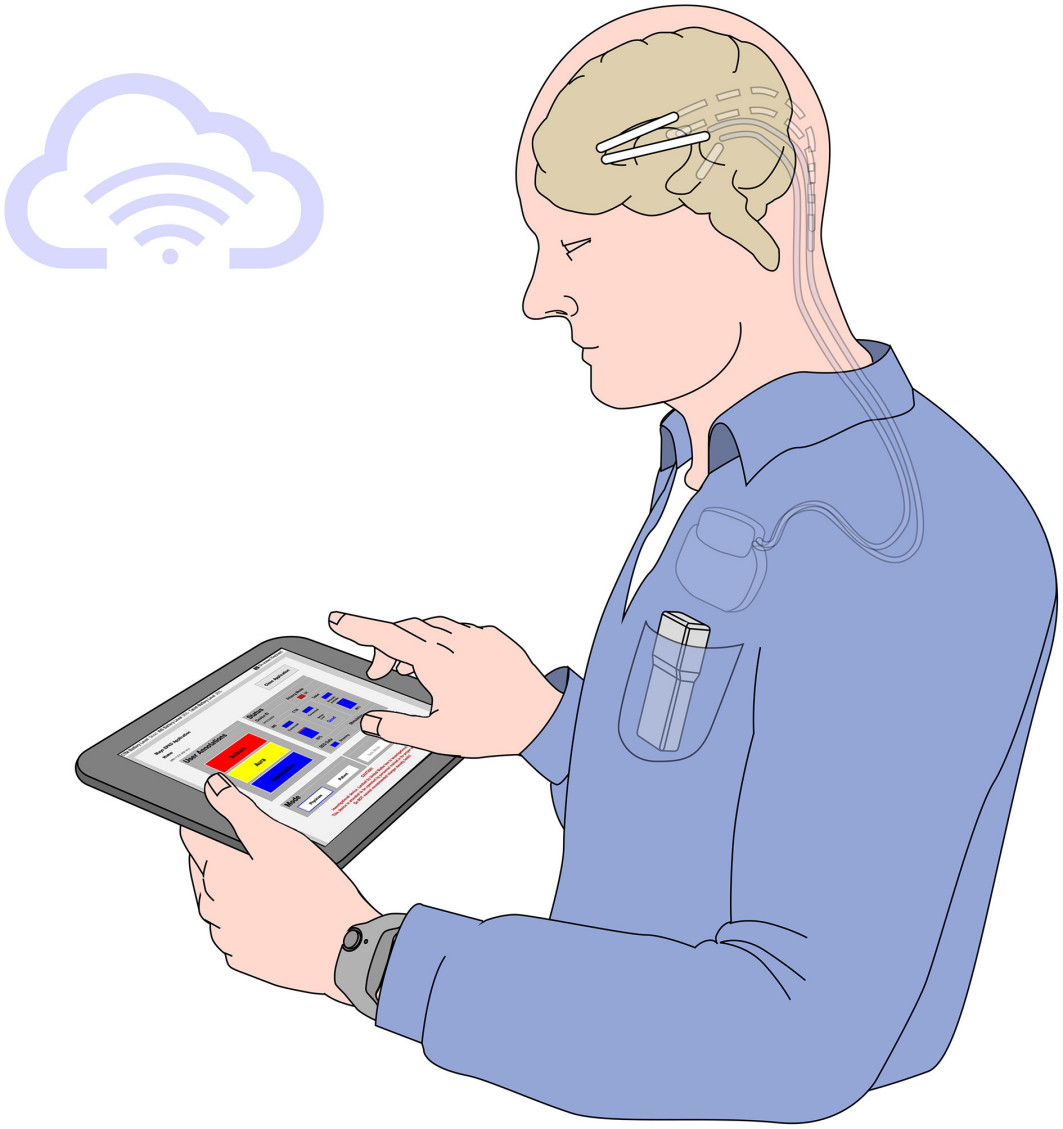
The EPAD system—The EPAD system user interface and core logic deployed along with on-tablet seizure detection and forecasting algorithms. EEG data packets from the investigational Medtronic Summit RC+S^TM^ implanted device are decoded, sorted, assembled, compressed, and stored in a cloud-synchronized repository in Multiscale Electrophysiology Format (MEF v.3.0). In addition, dense behavioral inputs from the patient interaction with the EPAD system and data from external wearable devices are synchronized over Wi-Fi or cellular data networks to a cloud-synchronized repository.

The EPAD system aims to record objective EEG data during seizures and modulate stimulation therapy based on seizure forecasts, which raises several important issues. First, forecasting algorithms are too compute-intensive to run on an implanted device, requiring data to be telemetered to an associated computer. Second, response times to stimulation require algorithms running with multiple response timescales, as seizure-responsive stimulation must act very quickly to abort a seizure. In contrast, seizure forecasts occur tens of minutes before a seizure allowing more time to adjust stimulation. Third, dynamic adjustment of stimulation requires that algorithm implementations are compliant with regulatory requirements for software development and are confirmed to be safe by extensive testing. The investigational Medtronic Summit RC+S^TM^ neuromodulation device offers a unique combination of near-real-time intracranial EEG telemetry, on-device analytics, and modulated stimulation therapy that could enable therapies not previously possible. The system can be configured using the Medtronic Summit libraries and API in custom software, enabling advanced features. The system is illustrated in Figure 1.

**Figure 2:**
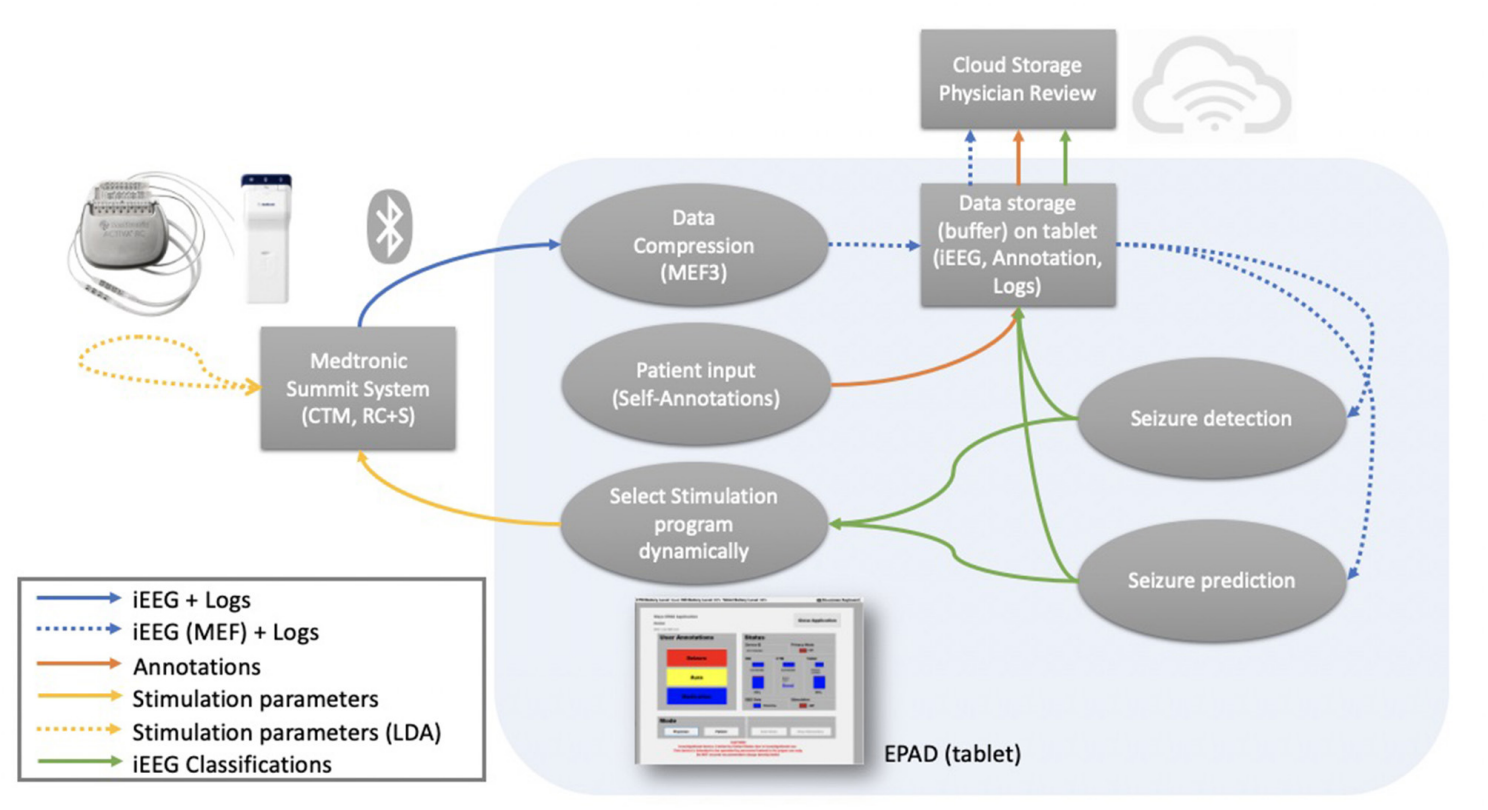
Data flow—Data flow between the different parts of the EPAD system. Colored arrows represent different data types flow. Blue (solid): data packets and logs from the from the investigational Medtronic Summit RC+S^TM^ are compressed. Blue (dashed): compressed iEEG data and logs are stored in a cloud-synchronized repository and used as input for the complex electographic seizure detection and prediction algorithms. Orange: patient-generated annotations are stored in a cloud-synchronized repository. Green: iEEG classifications from the electographic seizure detection and prediction algorithms are stored in a cloud-synchronized repository. Yellow (solid): stimulation parameters modulation based on EPAD iEEG classifications. Yellow (dashed): stimulation parameters modulation based on the embedded detector seizure detections.

The EPAD system user interface and core logic were developed in C#, and compiled python programs were used to deploy on-tablet seizure detection and forecasting algorithms. Data packets containing EEG and accelerometry from the implanted device are decoded, sorted, assembled, and losslessly compressed using Range Encoded Differences (RED) before being stored in a cloud-synchronized repository in Multiscale Electrophysiology Format (MEF v.3.0). Seizure, aura, medication, stimulation changes, and other event annotations are stored in SQL and CSV files. Video files acquired by the tablet's embedded camera during detected or self-reported seizures are synchronized with the EEG and accelerometry data and stored in the cloud repository. Dense behavioral input from the patient is received through interaction with the EPAD system, and data synchronization between devices, tablet, and cloud repository occurs over Wi-Fi or cellular data networks. Data flow between the different parts of the EPAD system is illustrated in Figure 2.

This architecture is beneficial for implementation using a mobile-computing middle layer responsible for configuring stimulation and sensing, managing data telemetry from the device to the cloud, and running moderately complex analytical algorithms in near real-time. This architecture allows the system to enable modulated therapy and provide objective EEG and behavioral data to physicians, patients, and caregivers.

## Design and Development

The EPAD system is designed to be used as part of an investigational device system early feasibility study to examine the safety and potential benefits of a novel closed-loop electrical stimulation therapy to treat drug-resistant epilepsy (DRE). The EPAD system tests the feasibility and potential benefits of three functions that may benefit the management of non-resectable DRE. (1) Seizure diaries arising from chronic electrographic recordings, analytics, and expert review may help physicians better manage patients' epilepsy. (2) Seizure forecasting, arising from predictive algorithms trained on physician-identified seizures, may allow patients to manage their epilepsy better. (3) Modulating stimulation based on electrographic biomarkers of sleep and seizures may enable the physician better to treat a patient's epilepsy with fewer side effects.

Software that is part of or controls a medical device is subject to strict regulation due to its safety-critical nature. Therefore, the EPAD system was developed following work instruction that meets the Food and Drug Administration (FDA) requirements for Investigational Device Exemptions (IDEs) as defined in 21 CFR 812.1 and Design Controls as defined in 21 CFR 820.30. In order to integrate traditional software development standards with the regulatory requirements, the EPAD system was developed with the V-model (20), a well-established software development life cycle model, also known as the Verification and Validation model. By its nature, the V-model is a good fit for many medical devices software development as the requirements is understood and clearly defined, and phases are complete one at a time with a testing phase at each step. That structure allows the detection of issues and inconsistencies in an early stage and is why the V-model is typically implemented within the field.

For the EPAD system, design and development were comprised of two main phases: Planning and Execution. The execution phase was further subdivided into Design, Implementation, and Delivery phases. The planning phase consisted of assessing stakeholder needs, performing a preliminary Safety Risk Analysis, and obtaining proponent review and acceptance of the proposed project plan. The execution phase was initiated with the design phase, where a System Requirements document was developed in consultation with physicians, patients, the Medtronic Summit System staff, and the Mayo design team. The System Requirements document captures the stakeholder needs translation into system requirements that describes the system's required functionality, performance, attributes, boundaries, and constraints. System Validation Test Protocol was developed during the design phase to define the necessary tests to ensure that the research system operates as intended in its operational environment according to the System Requirements document. During the development process's Implementation phase, the software and system requirements were translated into a working system and described in detail by the software design specifications. Finally, during the Delivery phase of the development process, the EPAD software was installed and configured on a tablet computer, and verification testing was performed. Residual system deficiencies were addressed, and final testing reports were generated, including the verification testing report and unresolved anomalies report.

The majority of the EPAD system's design effort consists of developing the EPAD software application and is detailed below.

### Medtronic Summit Research Development Kit

The Medtronic Summit System consists of a Model B35300R Olympus RC+S^TM^ implantable device (INS), commercial leads, and extensions, Model 97755 Charger, Summit Programming Application, Model 4NR010 Summit Research Lab Programmer (RLP), Model 4NR011 Continuous Telemetry Module Gateway (CTM), and Model 4NR009 Summit Patient Programmer. Stanslaski et al. (16) detail the two major parts of the investigational Medtronic Summit RC+S^TM^ system, including (1) an implantable hardware firmware subsystem for neural interaction and running embedded algorithms and (2) a supporting firmware-software system for communicating, recharging, streaming, and analyzing data. The EPAD system interaction with the investigational Medtronic Summit RC+S^TM^ systems enables the iEEG monitoring, processing, and control functions of the overall EPAD system. In addition to analysis on the electrographic signals to trigger changes in physician-defined safe neurostimulation approaches.

The Medtronic Summit Research Development Kit (RDK), a software interface library, was incorporated into the EPAD system and used to access gateway functionality. The RDK Library is a pre-compiled Dynamic-Link Library (DLL) file written in the C# programming language. All control and feedback functions with the investigational Medtronic Summit RC+S^TM^ INS must be handled through the API. Therefore, methods implemented in the RDK are called directly throughout the EPAD system. The Medtronic Summit RDK requires the Application Programming Interface (API) to run under Microsoft Windows. Hence, the EPAD system was developed as a mobile application capable of running on a Microsoft Windows Tablet computer.

The Research Lab Programmer (RLP) is the application/hardware the clinician uses to configure INS therapy safety-related settings and determine system status. Initial stimulation configuration, including contact selection and parameter limits, must be done on the RLP to ensure patient safety. After defining stimulation parameter space available with the RLP, the EPAD system can modulate stimulation within clinician-defined limits and modify other configurations using the Medtronic Summit API. However, it must adhere to the clinician-defined limits, or it will be rejected.

The Medtronic Summit API is used throughout the EPAD system to control and communicate with the entire Summit system and, specifically, the INS. This allows various actions, including initiating a link to the CTM and INS, device status queries, interactions logging, data streaming, and configuration of the device's sensing, data processing, classifiers, and adaptive control policy. The SummitManager enumerates and manages the telemetry to the implantable device. Using telemetry information, the SummitManager can create a Summit system object. The SummitSystem and SummitManager objects are vital objects within the Medtronic API to maintain the connection between the tablet, CTM and INS.

The EPAD application utilizes a 30-s keepalive timer active while running. The keepalive timer reads the CTM battery status and the INS battery status. These status queries are used to report status on the main screen and write hourly status annotations. Also, by querying the battery levels, we can ensure that a proper connection is maintained between the tablet, the CTM, and the INS. If more than three attempts to communicate with the CTM fail, then API commands can re-establish communication. The Medtronic API offers a callback function that signals the EPAD system when the connection is broken and should be re-established. When a new connection to the INS and CTM is needed, the keepalive timer re-initializes the data collection using a customized helper function. The function uses a Medtronic Summit API function to search over Bluetooth for known CTM devices, as only a CTM device previously paired with the tablet can be connected. Then uses a Medtronic Summit API function to discover and connect to the INS and an abstraction layer to manage all underlying functionality of a single INS.

The INS is queried to its state in terms of streaming data and stimulation. This information is used in additional features of the keepalive timer function, including a warning message that is displayed to the user when EEG data is expected, however, the tablet has received no data in 30 min. Also, the current set of stimulation parameters set on the INS are compared against desired stimulation parameters. If a discrepancy is found, the stimulation parameters are re-sent to the INS. These stimulation parameters, along with other basic setup parameters, are saved when any system parameter is changed, and hourly status updates of the entire system are generated. Furthermore, parameters, such as stimulation and LDA classifier settings, can have a daytime/nighttime mode to allow the patient to sleep better. This is also monitored and adjusted during the 30-s timer.

### MEF 3.0

Multiscale Electrophysiology Format (MEF) is an open-source file format designed to store electrophysiology and EEG (21) but is extensible to most time-series data. The format incorporates a header, which contains technical information about the file, stored data, and subject identifiers. The header is followed by a variable length sequence of compressed data blocks, each of varying size. Each compressed data block contains a block header, which contains the uUTC timestamp of the first data sample in the block, the number of samples stored in the block, a cyclic redundancy check value computed on the compressed data block, and a statistical model of the stored data samples. The format incorporates 128-bit encryption, which can be applied optionally to subject identifiers in the file header, technical acquisition details in the file header, and data blocks. Version 3.0 of MEF is designed to be a real-time data format, which means that a viewer/analyzer can read files even as another process is writing them. The MEF 3.0 file format is fully detailed in the Multiscale Electrophysiology File Format Version 3.0^1^.

The EPAD system receives data in packets from the Medtronic API callback commands. Packets typically represent either 50 or 100 ms with a corresponding timestamp. Upon receipt, packets are added to a buffer, which collects between 5 and 10 s of data, and sorts the packets into order using the given timestamp. Packets that are severely delayed (due to Bluetooth or other problems) and cannot be ordered correctly are discarded. Statistics of discarded packets are kept and monitored to ensure that data loss is not unreasonably high.

The MEF 3.0 API library (“meflib”^2^) is written in the c language and is a collection of ~100 functions designed to ease the use of the MEF 3.0 format. In addition, a secondary library (“mefwriter”^3^) is used to simplify the MEF 3.0 encoding process. This allows complete MEF 3.0 data channels to be encoded by using as few as three function calls, and most details of the file format are abstracted from view. In the EPAD system, both the meflib and mefwriter libraries are compiled as c functions and exported as dll files for use by the C# environment. This allows seamless integration with the EPAD system without having to recompile the libraries under C#.

MEF 3.0 supports segments, which allows EEG channels to be divided into arbitrary size files. The EPAD system takes advantage of this by beginning a new segment each time the application runs (typically daily, since EPAD software restarts at midnight each day). During the process of creating a new segment, the previous segment (and only the previous segment) is read and verified for consistency. This is necessary for both technical reasons (some data persists across segments) and guarantees that the on-board analytics algorithms can successfully read the entire channel. If even one segment were to be unreadable, then potentially, the entire channel might be unreadable. Since only the previous segment is read before creating a new segment, this allows much older segments to simply be deleted in the interest of reducing hard drive usage. The meflib library is sufficiently robust to merely ignore non-existent segments.

Analysis shows that using MEF 3.0 in the EPAD system, the EEG data (or time-domain data) is losslessly compressed at a ratio of better than 5 to 1, relative to the original JSON data produced by the Medtronic Summit API. This compressed MEF 3 version of the data is immediately indexed and readable using MEF 3.0 tools as the files continue growing in size. The compressed nature of the data represents less network traffic when uploading to the cloud and greater data storage capacity on the tablet hard drive. The uncompressed JSON version of the data is deleted every 24 h due to being redundant.

In addition to writing MEF 3.0 data, the EPAD system also uses a Python version of the MEF 3.0 library (“pymef”^4^) to read MEF 3.0 data that has already been written. This library consists of a Python interface designed around the native MEF c code compiled for Windows. As mentioned above, the EPAD system writes MEF 3.0 data, and data analysis is done in near real-time for seizure detection and prediction purposes in separate spawned processes that use the python MEF 3.0 reader library.

### Hardware Settings

The EPAD system can read or write INS settings using the various Medtronic API functions to manage the INS settings. There are write parameters functions to directly modify, change, or set various INS firmware settings and read parameters functions to access the INS state.

#### Sensing Parameters

The Summit system is sensing signals measured using the INS. The INS can stream up to four channels of local field potentials (LFPs) from the implanted electrodes. The EPAD system presents the physician with a series of configurable options representing the operation of the EPAD system and INS, including iEEG electrode configuration, sampling rates, sampling interval, duration, and accelerometer data telemetry from the INS. Initial selected values reflect options previously configured using the Medtronic Summit RLP.

A Medtronic API function is used to configure the sensing state and then sensing data that can be streamed from the INS to the EPAD system tablet. Individual streams can be enabled or disabled at any time. Each enabled data stream indicates the INS to send data packets, which is then handled on the EPAD system accordingly. The function first parameter is a Boolean, which determines if time-domain (TD) packets are sent from the INS/CTM to the tablet. The rest of the function Boolean inputs correspond to the following individual streams: Fast Fourier Transform (FFT) of the signal, Power (the input to the on-board classifier), Detection Events, Adaptive Stim (active state and stim settings), 3-axis Accelerometer data, Time-Sync (enables packet gen times), and Loop Recorder (LR) status updates and external marker echoing. General practice is not to request more information than is needed since the INS-CTM connection and the CTM-tablet Bluetooth connection have a data throughput limit.

The EPAD system is set by default to continuous real-time streaming but can also operate in periodic streaming. When designing the EPAD system, the design included putting the INS into a periodic streaming mode to save battery life, as it would only be streaming for 1 min out of every 3 (for example). However, it is necessary to close and reconnect the Bluetooth connection repeatedly to save a meaningful amount of battery power, and reconnection could be problematic if the CTM is not maintained in close proximity to the INS.

#### Stimulation Parameters

The ability to modulate stimulation based on patients' electrophysiological biomarkers, seizure diary and cyclical patterns holds unique potential for responsive and predictive adaptive neuromodulation. Modulating the intensity of stimulation based on electrophysiological biomarkers could allow applying high stimulation when a patient is at high risk of seizure and low stimulation when a patient is at low risk of seizure, by doing so reducing side effects and prolonging battery life. Also, neurostimulation intensity could be increased/decreased during sleep stages which a patient's seizure diary suggests a correlation with an increased/decreased likelihood of seizure occurrence. As well as, seizure diaries coupling to increase stimulation during sleep after seizures to disrupt memory consolidation and prevent the brain from strengthening seizure pathways (22).

To mitigate patient risk, the Medtronic API is limited by design in its ability to adjust therapy for a patient dynamically. Therefore, the EPAD system is limited to turning therapy on/off, switching between stimulation groups, and changing stimulation parameters within clinician-defined limits (configured with the RLP). The INS can be configured with up to four stimulation groups A, B, and C for general open-loop therapy and group D for adaptive stimulation.

A Medtronic API function is used to change the active group to a different one specified by the function argument. The EPAD system uses group A as a “safe mode” state where the baseline settings are used, when adaptive stimulation is turned off. Groups B and C are used for the stimulation trial. Finally, group D is used for adaptive on-board stimulation in combination with the classified state [baseline (wake and sleep), seizure, pre-seizure].

**Figure 3:**
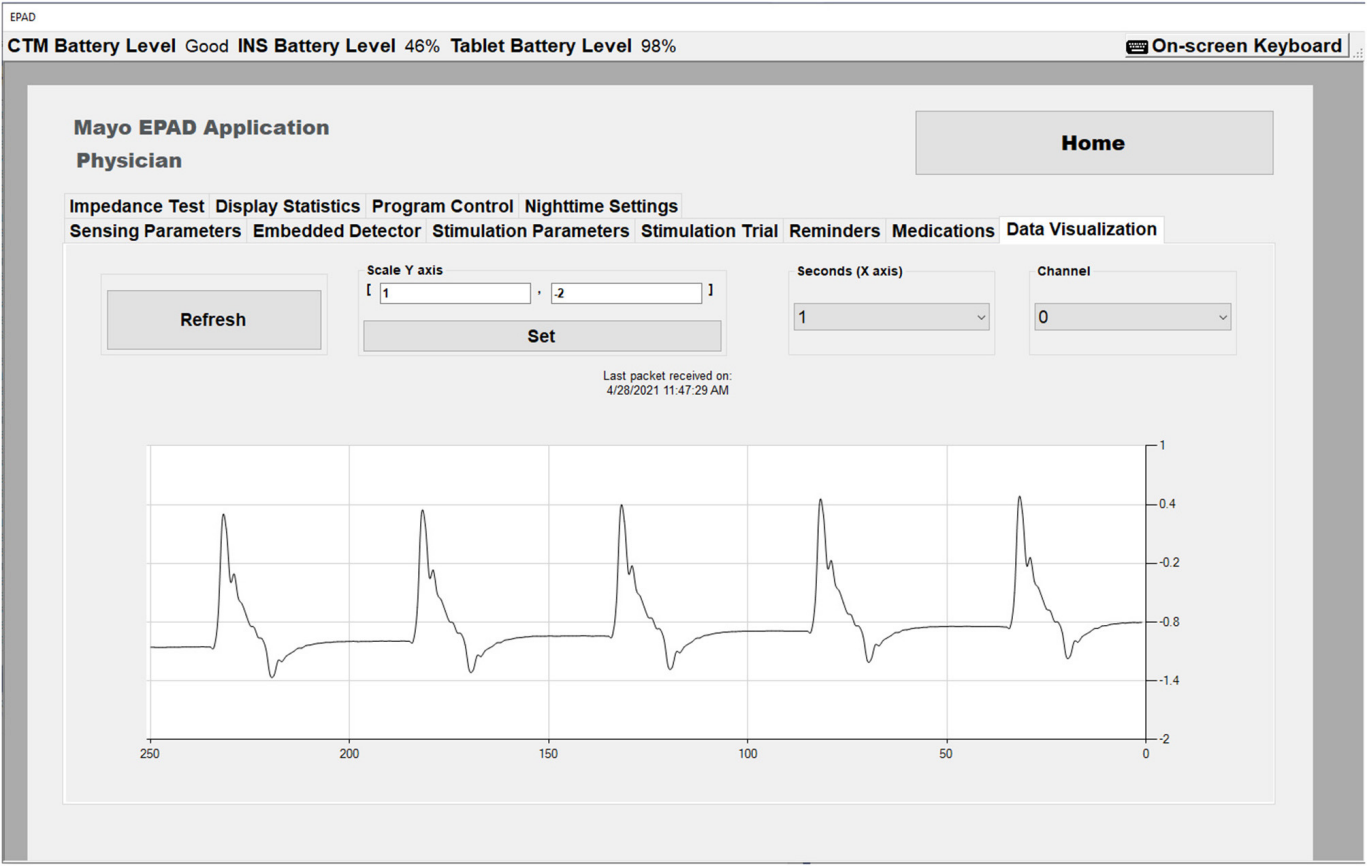
The EPAD Data Visualization tab in Physician mode showing real time data with stimulation artifact (Amplitude = 2 mA, Rate = 5 Hz).

The EPAD system presents the physician a set of configurable options affecting the stimulation modes, including stimulation rates and stimulation current amplitudes for each classified state [baseline (wake and sleep), seizure, pre-seizure]. When configuring an INS group program with the RLP, a clinician can define an upper and lower bound for each parameter. Because of the safety-critical nature of stimulation parameters effect on the patient, these are validated within the EPAD system against desired settings, clinician-defined limits and globally defined limits, every time new parameters are applied and within every EPAD system 30-s keepalive timer. When calling the API function that is used to turn on the stimulation engine and is required for any therapy to be output to the patient, additional validation occurs. The function will be rejected if there is no valid therapy configured or the INS has shutdown unexpectedly. The EPAD system includes a real-time data visualization capability that allows the physician to view the real-time effects of the configured stimulation parameters (Figure 3). This is a valuable tool giving the physician a unique view of the data immediately. The data visualization also assists in the Verification and Validation test.

The Summit system supports sensing-based adaptive algorithms, where the algorithm acts autonomously on the INS. Embedded adaptive therapy is only allowed in Group D. The system will be configured to operate with the on-device linear discriminant analysis (LDA) closed-loop classifier active, employing one LD with one threshold. It can have a value above the maximum threshold (High) and below the minimum threshold (Low). The embedded classifier will be configured to detect iEEG characteristics similar to physician-confirmed electrographic seizures and will implement physician-configured stimulation parameters (Amplitude and Stimulation Rate) in response to these detections. Using the embedded detector to detect known electrographic seizure events provides the fastest possible response to these iEEG changes, while maintaining reasonably high sensitivity and specificity.

#### Stimulation Trial Parameters

Finding the right set of stimulation parameters for each patient is complicated due to the time-consuming nature and the need to record real-word responses to the parameters. This feature allows the physician to pre-configure up to 24 parameter combinations, including stimulation amplitudes (3), rates (4), and pulse widths (2). Stimulation groups B and C are used for the stimulation parameters trial. Parameter groups are arranged in two sets, each of which can have unique pulse widths and electrode contacts. Within a set the parameter sequences occur in sequential combination so that every stimulation rate is tested with every set of amplitudes. The physician can specify the duration of each stimulation cycle as well as a “rest interval” between stimulation cycles. The stimulation trial can be repeated by selecting the number of test cycles and can be stopped at any point in the process while the tablet is connected. In case the software crashes or the tablet turns off during the trial, upon reconnection, the stimulation trial shall resume from the last set of parameters tested.

#### Impedance Measurements

The EPAD system can conduct an impedance measurement on up to 16 contact pairs in a test using an API function. The EPAD system warns the user that stimulation and sensing will be temporarily discontinued and waits for any active sensing loops to complete before turning off sensing and stimulation functions. Once these functions are confirmed to have stopped by the INS, the EPAD system conducts the impedance test. Measured impedances for electrode contacts shall be written to the log file and the Annotations file.

### User Interaction

Technology is advancing rapidly, and there are more and more digital personal assistants with advanced capabilities. Also, there are many new technologies for better diagnosis of diseases and better-targeted therapy to such conditions. The EPAD system combines these possibilities and provides an interface to allow the epilepsy patient to enter annotations regarding seizures, auras, and medications. The patient can make an annotation, by simply clicking the appropriate button on the main screen of EPAD. Once the patient presses the Seizure or Aura buttons, a video recording is started using both the tablet's front and back cameras, encrypted and saved in Audio Video Interleave (AVI) format. The EPAD system also provides the reminder feature, allowing the physician to pre-schedule reminders for medication dosages and battery charging. Reminders and alerts for INS, CTM, and Tablet batteries can be sent to the patient's smartphone via SMS text message. The physician medications tab allows the physician to enter the patient's medication dosages then automatically generates reminders for each dose. When the Medication button is pressed, a “medication dose” dialog is presented, which allows the patient to select the appropriate medication and dose taken. In the Patient-only section, an additional option is given for extra medications taken that are not part of the usual regimen specified by the physician.

Epilepsy is associated with sleep, cognitive, and psychiatric comorbidities, which significantly impact the quality of life. The EPAD system offers a unique opportunity for long-term tracking of cognitive performance and psychiatric symptoms using the questioners feature of the reminders that allow the physician to schedule mood survey questionnaires, including the IMS-12 (23) and surveys of premonitory symptoms of seizures (24).

All patient-generated annotations are displayed in the Patient Annotation Diary, and the physician has access to these annotations through synchronization of offline data files.

### Annotations

Prior studies have documented significant under-reporting of seizures in many patients (13). Chronic recordings may help physicians identify unreported seizures and adjust treatment accordingly. This could identify patients at significant risk of status epilepticus or SUDEP. Also, it may be possible to identify circadian or ultradian patterns in a patient's seizures and use these patterns to optimize therapy (25). Additionally, chronic monitoring and seizure diaries could provide a clearer view of a patient's seizure patterns and suggest a potential resection target not apparent upon initial monitoring. Many patients with intractable epilepsy also exhibit behavioral, non-epileptic spells. Therefore, objective recordings may help physicians differentiate among conflicting patient reports or evidence and provide an alert for physicians or caregivers in the event of prolonged seizures or status epilepticus.

To investigate the potential benefits of an electronic seizure diary, we incorporated the ability for seizure annotations within the system to be generated by the patient or generated by automated seizure detection algorithms. In addition to user interface interactions, a back-end database is updated with auto-generated events. Events are notated in two different ways to allow greater flexibility upon reading. First, through the use of a simple text file in Comma-Separated Value (CSV) format, and second, in a relational database structure using Structured Query Language (SQL). In the interest of being lightweight and not computationally intensive, the EPAD system makes use of SQLite, which is an embedded, open-source c-language-based SQL management system. The resulting csv and sqlite files are synchronized to the Mayo BNEL lab's servers via Dropbox.

Besides the patient-generated events, numerous system status events are also incorporated into these files. These include embedded (INS) detections of seizure-like electrographic anomalies, on-tablet detections of seizure-like electrographic anomalies, on-tablet computed pre-seizure state warnings, battery levels for INS, CTM, and tablet computer, stimulation pulse rate, amplitudes, channels, and pulse width when configured, and on/off states, timestamps, and settings for detectors, stimulation, accelerometer, and other signals saved. These parameters are updated at least hourly and upon predefined trigger events to give the physician a complete picture of the EPAD system's configuration at any point in time.

### Data Analysis

Reliable seizure forecasting holds great benefits to the patient, including permitting patients to take fast-acting medications to prevent seizures and also may improve patient safety by allowing patients to avoid potentially hazardous activities during high seizure likelihood periods. Additionally, seizure forecasting may reduce psychiatric comorbidities of epilepsy by reducing the anxiety and depression associated with seizure unpredictability. Moreover, seizure forecasting could enable increasing the intensity of neurostimulation during seizure-prone periods to prevent seizures.

Beyond the embedded detector used to detect electrographic seizure events that provide the fastest possible response, the EPAD system also runs more complex analytic algorithms to detect iEEG changes preceding physician-confirmed electrographic seizures, and iEEG changes associated with physician-confirmed wake and sleep states. The algorithms for classification of brain state (e.g., seizure, pre-seizure, wake, sleep, epileptiform activity) from acquired iEEG data have been developed in Python and compiled for Windows. The algorithms are trained offline and algorithm parameters are loaded to the tablet after training. The main EPAD program calls the compiled Python executable files through the Windows file system with the necessary parameters. Based on these iEEG classifications, the EPAD System will change the baseline stimulation parameters to physician-defined values designed to optimize neuromodulation therapy for particular brain states.

#### Electrographic Seizure Detection

The role of EPAD system seizure detection algorithm is to support a seizure diary of physician-confirmed electrographic seizures. The EPAD system implements a modified version of the best performing seizure detection algorithm from the recent machine learning seizure detection contest (26) to accommodate continuous iEEG data. The algorithm was initially developed for the competition, used a Random Forest classifier (3,000 trees) with frequency spectrum, time-domain correlation, and frequency-domain correlation features. Specifically, the algorithm aggregates the logarithm of the Fourier transform from 1 to 47 Hz (in 1 Hz bins), the correlation coefficients, and eigenvalues of the correlation matrix between Fourier transformed iEEG channels from 1 to 47 Hz, and the correlation coefficients and eigenvalues between the raw iEEG channels. This algorithm's EPAD system implementation uses the same feature set described above, with a reduced number of 150-tree Random Forest classifier, operating on 1-s iEEG data segments.

#### Electrographic Seizure Prediction

The seizure prediction algorithm's role is to support stimulation parameters modulation in periods preceding physician-confirmed electrographic seizures. It is reported in the literature (13, 14) and supported by our internal testing that these identifiable iEEG signatures occur 60 or more minutes before the onset of a seizure. The EPAD system implements a modified version of the best performing seizure prediction algorithm from a recent machine learning seizure prediction contest (27), to accommodate continuous iEEG data. The algorithm was initially developed for the competition used a cross-validation strategy to select the best performing classifier among Logistic Regression, Linear Regression, and Support Vector Machine classifiers, and used a genetic algorithm (or random index) to select a subset of features from among the following: Time correlation matrix upper right triangle and sorted eigenvalues, frequency correlation matrix upper right triangle and sorted eigenvalues, the logarithm of the FFT magnitude for various frequency ranges, power-in-band spectral entropies, Higuchi fractal dimension with Kmax of 2, Petrosian fractal dimension, and Hurst exponent.

The code developed for the contest was modified to run with iEEG data from the EPAD system and simplified to improve execution time. Preliminary algorithm testing on canine iEEG recordings revealed that the Logistic Regression classifier was the most reliable algorithm, and that a vastly reduced feature set was adequate to achieve good performance. The EPAD implementation of this algorithm uses the Logistic Regression classifier, operating on 10-min iEEG data segments (28). The feature set was reduced to interelectrode correlation and the FFT magnitude between 0.25 and 24 Hz. The prediction algorithm was deployed as a separate executable, so that future advances in prediction algorithms could be incorporated without recoding the core application.

### Risk Analysis

The EPAD system was developed with a test-driven evolutionary development strategy. We conducted an analysis of risks associated with the use of the EPAD system and evaluated these risks using an acceptability framework defined by our institution's Division of Engineering. This analysis of risks includes a summary of the overall residual risk and the acceptability of residual risk levels that have been attained through specific risk mitigations. The main residual risks of the EPAD system include loss of therapy due to battery depletion in the implanted neurostimulator, changes in electrode impedances over long time periods decreasing effectiveness of sensing and stimulation, electromagnetic noise triggering high stimulation states when not intended, and unsafe stimulation programs being implemented by the physician.

A definition of Essential Performance is established, as well as the safety classification of software components used as part of the device system. AAMI/IEC 60601-1 defines Essential Performance to be “Performance necessary to achieve freedom from unacceptable risk.” The standard notes that “Essential Performance is most easily understood by considering whether its absence or degradation would result in an unacceptable risk.” Risk analysis suggests that the Essential Performance of the EPAD is to notify the user of its operational status. Because it operates via an external connection to the Medtronic Summit System, failure of the EPAD system to operate would not interfere with the Summit System's ability to deliver stimulation. “Safety Classification” is used to establish the classification of the software component and is used to inform software development and verification efforts. Software safety classes include: Class A: No injury or damage to health is possible, Class B: Non-serious injury is possible, and Class C: Death or serious injury is possible. The software components related to iEEG recording are found to be Class A, while the stimulation modulation is found to be Class C; the system as a whole is considered Class C.

### Verification and Validation

**Figure 4:**
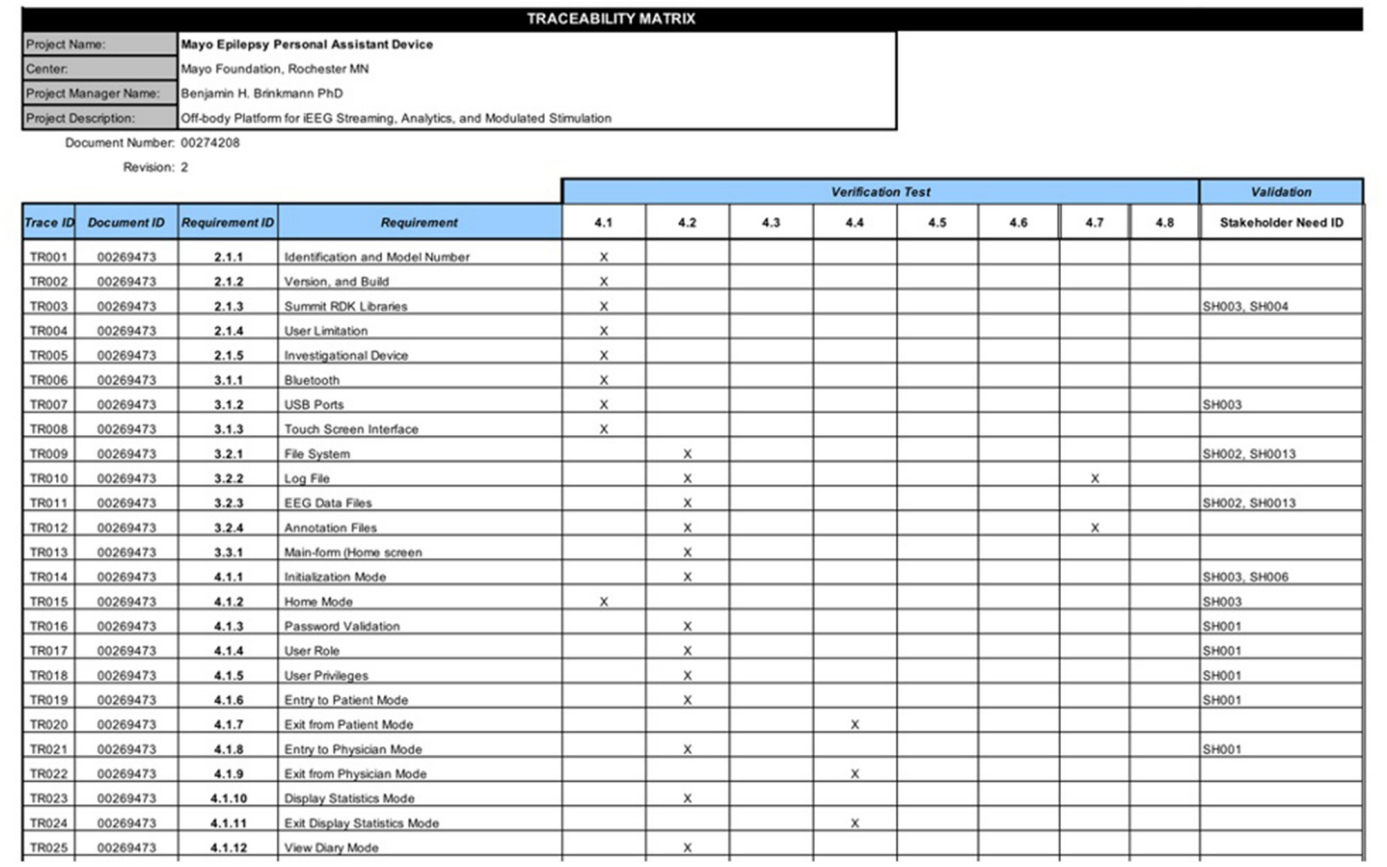
An excerpt from the EPAD Requirements Traceability Matrix.

Verification and validation testing are done to confirm that a device and software meets its design specifications and if it fulfills its intended purpose. An essential component in a quality management system is the Requirement Traceability Matrix which is shown in Figure 4. The Requirement Traceability Matrix links verification tests to the individual design requirements they test, and similarly validation tests to user needs. This ensures that all necessary design inputs are addressed by the design and confirmed by testing. Best practices in software verification include embedded and manual build-time testing. With every new build of an EPAD version, we performed manual tests designed to cover broad use cases and reveal deficiencies quickly broadly. Unit testing of individual software elements is also part of best practices in software design and was employed throughout the EPAD code base and performed automatically with each build. Unit testing generates pass/fail reports for each test, and these are logged and reported in the Verification Testing Report. A comprehensive verification testing plan was followed which specified the testing environment, the materials needed, and the pass/fail criteria for the test, in order to verify that requirements defined for the EPAD had been implemented successfully. A vital element of the EPAD system is the integration of several independent software components. For that reason, verification of successful component integration was explicitly tested as part of the verification test plan. Testing integration of modules within the code was handled using bottom-up automated testing at build time, with integration tests written to confirm the correct cross-module operation. Specifically, the interactions with the main EPAD code and the Medtronic Summit RDK, Seizure Detection Algorithm, Seizure Prediction Algorithm, and MEF Library were confirmed. Finally, validation tests were performed according to a detailed testing protocol to confirm the design and implementation met the input stakeholder needs. All testing results were carefully documented and stored in a document control system (SolidWorks PDM, 3DS Inc., Waltham MA).

Tables 1, 2 summarizes some of the basic verification and validation tests used in the EPAD quality system. Testing was performed on a dedicated benchtop system or in preclinical canine studies in a colony of research dogs with epilepsy (29). A particular challenge in testing this type of application is the requirement to test detection algorithms over a large number of seizures. Doing this sort of testing in real-time in canines would require a prohibitively long time to accomplish, and it was necessary to use some creativity in devising achievable tests that would confirm the system's performance. Availability of both benchtop INS systems and implanted canines was essential for system design and testing. While some tests could be performed in both environments, many required one or the other.

**Table 1:** Verification tests used in the EPAD quality system.

**Requirement**	**Test**
EPAD shall disconnect when the INS battery reaches 25% to prolong battery life and prevent loss of therapy	Canine subject's INS battery was partially charged, and EPAD disconnection was observed when it reached 25% power
The embedded LDA detector shall identify at least 80% of physician identified electrographic seizures with a false positive rate of <20%	The benchtop device is attached to electrodes immersed in a saline bath. EEG signals previously recorded from canines with epilepsy were electrically conducted into the saline bath using an Arduino. EPAD recorded the EEG and LDA seizure detections, and these detections were compared to the canine EEG signal annotations
The Application shall modulate the amplitude and frequency of stimulation in response to the output of iEEG analytics, with frequencies and amplitudes as configured by the physician. iEEG analytics shall identify iEEG characteristics similar to data preceding physician-identified seizures (pre-ictal)	Phase I: The python executable performing seizure prediction was trained on retrospective canine iEEG data and tested on over 60 days of data on a separate computer to verify performance Phase 2: The same executable running on the tablet computer as part of EPAD was trained to identify delta wave sleep and to initiate very low amplitude (0.5 mA) stimulation on a canine subject. Recorded iEEG data was reviewed to confirm stimulation artifact was visible during delta wave sleep
EPAD shall provide the ability to conduct a stimulation trial, cycling through at least 12 sets of stimulation amplitudes and frequencies on up to 2 sets of electrodes	Stimulation trial was configured with notably different amplitudes and frequencies on different electrodes. The stimulation trial was run first on the benchtop device and then on a canine subject's device with EEG recording enabled. Stimulation artifacts on recording electrodes were used to confirm relative stimulation rate and amplitude changes

**Table 2:** Validation tests used in the EPAD quality system.

**User need**	**Test**
Ensure the EPAD system initializes a connection to the Medtronic Summit System if available	Medtronic INS and CTM initially paired with EPAD system is moved out of range (>2 m) until connection drops. When moved back within range the system initiates a wireless connection within 60 s
Ensure the EPAD system can provide near real-time EEG data display	With a benchtop device paired, the user navigates to the EPAD Data Display tab, which provides near real-time display of captured iEEG data. While watching streaming iEEG data, the user taps the electrodes and confirms that high amplitude artifacts appear in the display within a few seconds
Ensure the EPAD system buffers acquired data locally if no network data connection is available	With a benchtop or canine EPAD system the user disconnects from Wi-Fi networks and enables iEEG streaming for 24 h. The user confirms that iEEG data files from the disconnected day are stored on the tablet and that iEEG files are transferred once Wi-Fi is re-established
Ensure the EPAD can provide reminders, queries, and questionnaires to the patient	The EPAD system was configured to provide notifications via dialog windows and SMS notifications for medications, mood surveys, and battery charging. Notifications of each type were set to occur at 5-min intervals over the course of a few hours with SMS messages directed to the user's phone

### Analytical Platform and Data Visualization

**Figure 5:**
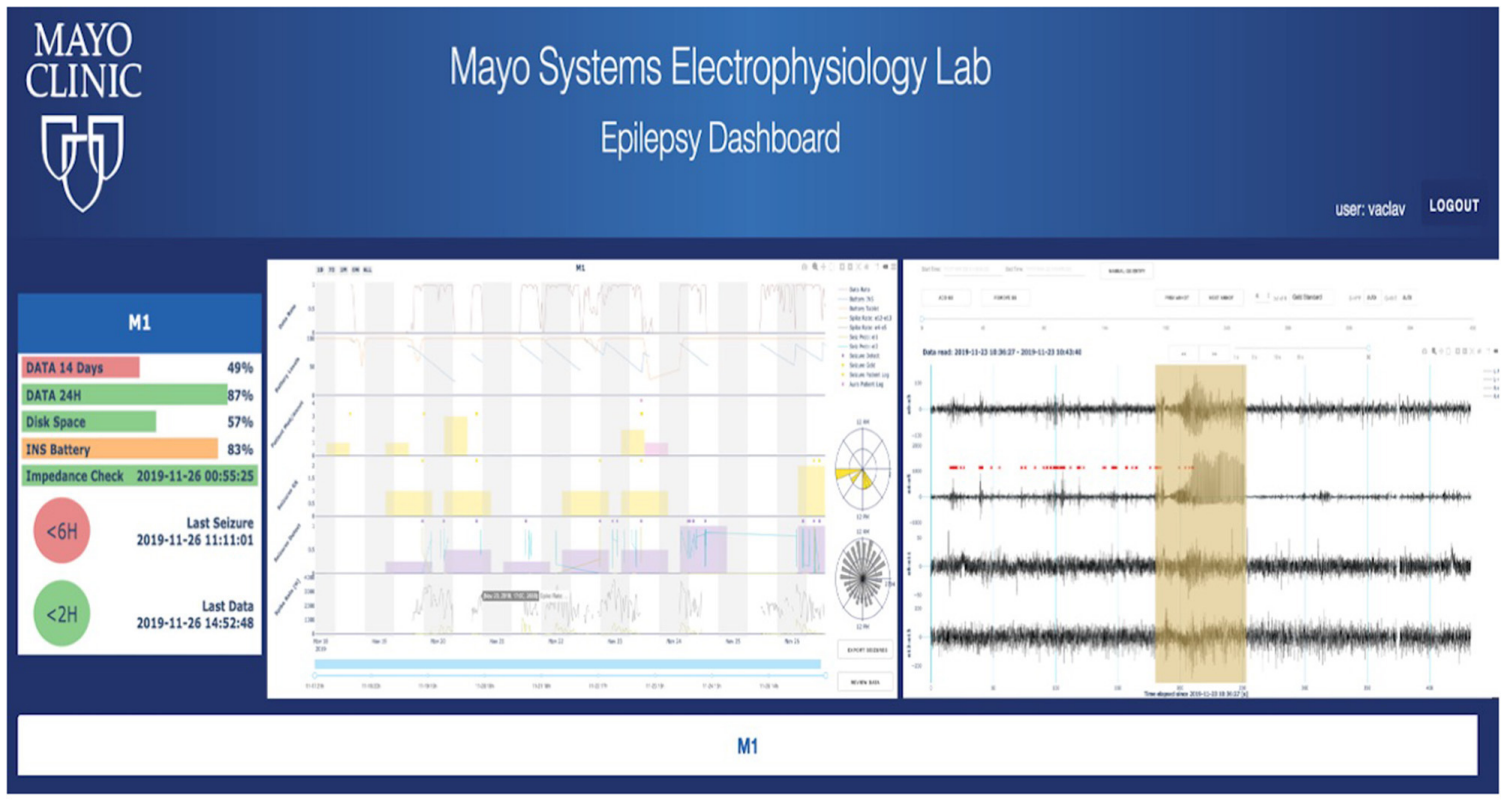
Cloud analytics—First human subject data in the cloud longitudinal analytics system, including automated seizure detections and gold standard (expert reviewed) annotations in raw iEEG data. Other features of the iEEG are displayed, such as spike rates and their circadian timing.

To take advantage of rich data streamed from the EPAD system, the analytical backend and the cloud-based physician Epilepsy Dashboard provide a platform for reviewing electrophysiology data wirelessly telemetered off the implant. The data are automatically processed with a battery of algorithms running on the patient's local handheld for detecting seizures, IES, and classifying sleep/wake behavioral state. Results are stored in a database and accessible via a web-based dashboard. The Epilepsy Dashboard enables swift review of immediate and long-term data trends from the device (e.g., battery, electrode impedances), electrophysiology data, and patient inputs. The physician can quickly review and either confirm or reject automatically detected, and patient reported events. An example of the Epilepsy Dashboard is shown in Figure 5.

## Discussion

The integration of implanted neuromodulation devices, external wearable sensors, and dense behavioral sampling with cloud data storage and computational capabilities represents a potentially transformative advancement toward fully integrated digital medicine. The EPAD system, in its role of collecting data and interacting with patients, facilitates new scientific investigations which would not otherwise be possible. The EPAD system was tested and validated in preclinical studies in canines with epilepsy (29) including pet dogs living at home with their owners, and has currently been used in three human subjects with drug-resistant epilepsy. In addition, the EPAD system plays a vital role in many of our group's ongoing projects. To investigate circadian and multidian cycles our group characterized these in 16 dogs with naturally occurring focal epilepsy that were continuously monitored with the investigational Medtronic Summit RC+S^TM^ combined with the EPAD system (10). This study shows that seizure timing in dogs with naturally occurring epilepsy is not random, and that circadian and multiday seizure periodicities, and seizure clusters are common. In addition, circadian, 7-day, and monthly seizure periodicities occur independent of antiseizure medication dosing, and these patterns likely reflect endogenous rhythms of seizure risk. The EPAD system was also used to improve seizure detection algorithm on-board of the investigational Medtronic Summit RC+S^TM^ device as the on-board detector offers the fastest possible response to iEEG changes, while maintaining reasonably high sensitivity and specificity. In this study, our group developed an algorithm using two power-in-band features with the on-board linear discriminant classifier to distinguish between seizure and non-seizure states. This simple algorithm can be implemented on the investigational Medtronic Summit RC+S^TM^ combined with the EPAD system and showed promise for detecting seizures recorded with leads in bilateral hippocampus (HC) and anterior nucleus of the thalamus (ANT). To investigate comorbid psychiatric disorders that are very common in drug-resistant epilepsy our group used the investigational Medtronic Summit RC+S^TM^ combined with the EPAD system to underline connections between epileptiform activity, mood, and therapeutic deep brain stimulation. Finally, in our group recent work we described the first application of a distributed brain co-processor, made possible by the EPAD system, providing an intuitive, bi-directional interface between device and patient, and implement it with human and canine epilepsy patients in their natural environments (30). Different algorithms, including automated behavioral state (wake and sleep) and electrophysiologic biomarker (interictal epileptiform spikes and seizures), were first developed and parameterized using long-term retrospective data from 10 humans and 11 canines with epilepsy and then implemented prospectively in implanted co-processors for two pet canines and one human with drug-resistant epilepsy as they live naturally in society.

The challenges remaining in remote monitoring and dynamic neuromodulation are primarily engineering challenges associated with battery life and reducing the overall burden of the system on patients. The success to date of the EPAD system serves as proof of feasibility, and we anticipate new systems with these capabilities will emerge in coming years. The EPAD and associated cloud infrastructure are applicable to other emerging devices (31–34), and continued development will allow integration of different physiological data streams in near real time from remote subjects.

The need for fully integrated remote monitoring and neuromodulation systems in clinical epilepsy is clear, and these systems have the potential to help address significant problems in epilepsy, including seizure under-reporting (35, 36), seizure forecasting (13, 37), psychiatric and cognitive comorbidities (38–40), and the lengthy duration currently needed to adjust neuromodulation parameters to optimize seizure control (6). Furthermore, this infrastructure facilitates new scientific insights into long-term patterns and trends in human and animal neurophysiology (9, 10, 41).

Important scientific investigation often requires innovative engineering solutions to enable measurement, study, and data gathering in ways not previously possible. The investigational Medtronic Summit RC+S^TM^ and EPAD systems are examples of cutting-edge engineering that enable continuous iEEG data collection for months to years across the full range of normal and pathological brain states, in concert with associated mood and symptom information, along with a full range of stimulation paradigms. The data accessible via this system is unparalleled and has the potential to transform neuromodulation therapy for epilepsy, mood disorders, and other conditions, and to advance our basic understanding of neuronal ensembles and local field potentials.

## Data Availability Statement

The data analyzed in this study was subject to the following licenses/restrictions: human data are restricted by patient privacy and HIPAA considerations. Requests to access these datasets should be directed to brinkmann.benjamin@mayo.edu.

## Ethics Statement

The studies involving human participants were reviewed and approved by Mayo Clinic Institutional Review Board. The patients/participants provided their written informed consent to participate in this study. The animal study was reviewed and approved by Mayo Clinic Institutional Animal Care and Use Committee. Written informed consent was obtained from the owners for the participation of their animals in this study.

## Author Contributions

TPA, DC, and BB drafted the manuscript. All authors contributed to editing, collecting, and analyzing data.

## Conflict of Interest

BB and GW declare licensed IP to Cadence Neuroscience Inc. GW has equity in NeuroOne Inc. MS was employed by the company DarkHorse Neuro. Medtronic Inc. supplied devices for this study at no charge and reviewed this manuscript for accuracy related to their device, but did not edit results or conclusions. The remaining authors declare that the research was conducted in the absence of any commercial or financial relationships that could be construed as a potential conflict of interest.

## Publisher's Note

All claims expressed in this article are solely those of the authors and do not necessarily represent those of their affiliated organizations, or those of the publisher, the editors and the reviewers. Any product that may be evaluated in this article, or claim that may be made by its manufacturer, is not guaranteed or endorsed by the publisher.

## Footnotes

^1^ https://msel.mayo.edu/files/codes/MEF%203%20Specification.pdf

^2^ https://github.com/msel-source/meflib

^3^ https://github.com/msel-source/mefwriter

^4^ https://github.com/msel-source/pymef
